# Mechanisms Employed by *Escherichia coli* to Prevent Ribonucleotide Incorporation into Genomic DNA by Pol V

**DOI:** 10.1371/journal.pgen.1003030

**Published:** 2012-11-08

**Authors:** John P. McDonald, Alexandra Vaisman, Wojciech Kuban, Myron F. Goodman, Roger Woodgate

**Affiliations:** 1 Laboratory of Genomic Integrity, National Institute of Child Health and Human Development, National Institutes of Health, Bethesda, Maryland, United States of America; 2 Departments of Biological Sciences and Chemistry, University of Southern California, Los Angeles, California, United States of America; Université Paris Descartes, INSERM U1001, France

## Abstract

*Escherichia coli* pol V (UmuD′_2_C), the main translesion DNA polymerase, ensures continued nascent strand extension when the cellular replicase is blocked by unrepaired DNA lesions. Pol V is characterized by low sugar selectivity, which can be further reduced by a Y11A “steric-gate” substitution in UmuC that enables pol V to preferentially incorporate rNTPs over dNTPs *in vitro.* Despite efficient error-prone translesion synthesis catalyzed by UmuC_Y11A *in vitro*, strains expressing *umuC*_Y11A exhibit low UV mutability and UV resistance. Here, we show that these phenotypes result from the concomitant dual actions of Ribonuclease HII (RNase HII) initiating removal of rNMPs from the nascent DNA strand and nucleotide excision repair (NER) removing UV lesions from the parental strand. In the absence of either repair pathway, UV resistance and mutagenesis conferred by *umuC*_Y11A is significantly enhanced, suggesting that the combined actions of RNase HII and NER lead to double-strand breaks that result in reduced cell viability. We present evidence that the Y11A-specific UV phenotype is tempered by pol IV *in vivo*. At physiological ratios of the two polymerases, pol IV inhibits pol V–catalyzed translesion synthesis (TLS) past UV lesions and significantly reduces the number of Y11A-incorporated rNTPs by limiting the length of the pol V–dependent TLS tract generated during lesion bypass *in vitro*. In a *recA730 lexA*(Def) Δ*umuDC* Δ*dinB* strain, plasmid-encoded wild-type pol V promotes high levels of spontaneous mutagenesis. However, *umuC*_Y11A-dependent spontaneous mutagenesis is only ∼7% of that observed with wild-type pol V, but increases to ∼39% of wild-type levels in an isogenic Δ*rnhB* strain and ∼72% of wild-type levels in a Δ*rnhA* Δ*rnhB* double mutant. Our observations suggest that errant ribonucleotides incorporated by pol V can be tolerated in the *E. coli* genome, but at the cost of higher levels of cellular mutagenesis.

## Author Summary

*E. coli* pol V, a complex formed by *umuC* and *umuD* gene products, is a “founding” member of the Y-family of DNA polymerases that have been identified in all domains of life. The primary cellular function of Y-family polymerases is the replication of damaged DNA. We discovered that pol V is characterized by unusually poor sugar selectivity and readily incorporates ribonucleotides into DNA. The extent of ribonucleotide incorporation can be modulated by substituting amino acids at, or adjacent to, the “steric gate” in the active site of the DNA polymerase. Principally, by taking a genetic approach, supported by *in vitro* biochemical data, we show that SOS mutations triggered by pol V–catalyzed errant ribonucleotide incorporation are kept in check by the action of nucleotide excision repair operating in conjunction with RNase HII and, unexpectedly, by another error-prone Y-family polymerase, pol IV. Our studies provide new insight into a growing field investigating the processing of ribonucleotides that are misincorporated by DNA polymerases and how these basic mechanisms contribute to cell survival and mutagenesis.

## Introduction

Translesion synthesis (TLS) allows living organisms to tolerate DNA damage to their genome. The vast majority of TLS in *Escherichia coli* is catalyzed by the LexA-regulated damage-inducible polymerases II, IV and V, which alone, or in various combinations, are recruited to the sites of DNA damage [1]. The B-family pol II which is encoded by the *polB* gene, is a rare case of a specialized TLS polymerase possessing 3′-5′ exonuclease activity [2]. As a result, pol II-dependent replication of both undamaged and damaged DNA is quite accurate with the exception of an N^2^-acetylaminofluorene adducts, where it promotes −2 frameshifts [3]. Y-family polymerases, pol IV, encoded by the *dinB* gene [4], [5], and pol V, the product of the *umuC* and *umuD* genes [6], are devoid of exonucleolytic proofreading and are characterized by low-fidelity DNA synthesis on undamaged DNA [7], [8]. Nevertheless, pol IV is remarkably accurate when replicating past certain DNA lesions, such as N^2^-dG adducts [9]. While pol II and pol IV each appear to facilitate TLS of a narrow range of damaged substrates, pol V is able to accommodate a diverse spectrum of DNA lesions in its active site and bears the greatest burden of TLS in *E. coli* [1], [6], [10]. Pol V-dependent TLS is highly error-prone causing the majority of cellular mutagenesis after DNA damage [6], [11].

Pol V, a heterotrimeric UmuD′_2_C complex [12], requiring the presence of a RecA nucleoprotein filament (RecA*) for optimal activity [13]–[17], has intrinsically low base substitution fidelity [18], [19]. We have recently discovered that this polymerase is also characterized by substantially reduced sugar selectivity [20]. When the canonical Watson-Crick base pairing is preserved, purified pol V accompanied by accessory proteins readily incorporates all ribonucleotides (ribonucleoside monophosphates, rNMPs) except uracil and catalyzes efficient and highly processive RNA synthesis *in vitro* in the presence of all four rNTPs. The ability of pol V to incorporate ribonucleotides is dramatically enhanced by a Y11A substitution at the conserved steric gate residue of UmuC, and greatly reduced by an F10L substitution [20]. In contrast, a Y11F substitution affects sugar selectivity minimally [20]. All three alleles also have different effects on base substitution fidelity and TLS activity of the mutant polymerases. Because the Y11F mutant readily accommodates G∶T mispairs in the active site, it induces higher levels of mutagenesis than wild-type pol V [20], but the ability of the wild-type polymerase and Y11F mutant to replicate damaged DNA is similar. The F10L_UmuC variant is characterized by a significant increase in the accuracy of nucleotide incorporation and moderate decrease in TLS activity. Consistent with this observation, cells expressing the F10L mutant exhibit low levels of UV-induced mutagenesis [21]. In contrast, the *in vivo* phenotype of strains expressing pol V with the *umuC*_Y11A substitution contradicts its *in vitro* biochemical properties. UmuC_Y11A is highly inaccurate *in vitro*, yet exhibits low mutability *in vivo* [20], [21]. Furthermore, despite the observation that the UmuC_Y11A variant catalyzes TLS past a T-T cyclobutane pyrimidine dimer (CPD) *in vitro* at least as efficiently as the wild-type enzyme, it confers minimal UV-resistance to a Δ*umuDC* strain [21]. To explain these phenotypes, we suggest that the dramatic increase in rNMP incorporation promoted by UmuC_Y11A leads to the induction of downstream pathways involving rNMP processing. Presumably the rNMP-targeted repair pathways would not only reduce *umuC*_Y11A-dependent spontaneous and UV-induced mutagenesis, but also interfere with completion of TLS resulting in the observed decrease in UV resistance.

Recent studies have demonstrated that similar to pol V, various DNA polymerases are able to incorporate ribonucleotides into DNA although in most cases less efficiently (reviewed in [22]). Even replicative polymerases with much more rigorous steric exclusion mechanisms insert rNMPs in much higher amounts than it was previously assumed [23]. In addition to misinsertion during replication or repair, stable incorporation of rNMPs in the DNA backbone could result from the incomplete removal of RNA primers used during maturation of lagging-strand Okazaki fragments. Due to the presence of a reactive 2′ hydroxyl on the ribose ring, rNMPs embedded in genomic DNA could sensitize the DNA strand to spontaneous and enzymatic hydrolytic cleavage. They can also cause distortion to the structure of the double helix that disrupts the ability of DNA-binding proteins to recognize DNA, thereby interfering with subsequent replication and transcription processes. Therefore, efficient repair of RNA/DNA mismatches is a critical process for a living cell, so as to ensure maintenance of genome integrity and, ultimately, its viability. As a result, cells have evolved various pathways for recognizing and removing aberrant rNTP incorporated into DNA strands [24]–[27].

The major enzymes initiating this pathway are ribonucleotide-specific endonucleases, Ribonucleases H (RNases H), which are present in organisms across all domains and are classified as types 1 and 2 based on sequence conservation and substrate preference [28]. Ribonucleases of both types are structurally related and have a similar mechanism of hydrolysis. However, while RNase HI cleaves the RNA moiety in the RNA/DNA hybrids with more than four sequential rNTPs embedded in a dsDNA strand, RNase HII enzymes can hydrolyze all kinds of hybrids, but prefer those which have a single rNTP embedded in DNA, rather than RNA/DNA duplexes with a stretch of riboses [29]. Although it is well established that RNase HI and HII are important for the release of rNMPs from a DNA duplex, the precise pathway initiated by these enzymes remains elusive. Based on *in vitro* studies, a general model describing the sequence of events that leads to the replacement of the ribose with deoxyribose has been developed for eukaryotic system. According to this model, after the phosphodiester bond of the nucleotide 5′ to the RNA-DNA junction is nicked by RNase H, an enzyme with 5′ to 3′ exonuclease activity makes a single cut 3′ to the rNTP, thus releasing the monoribonucleotide. After dissociation of the cleaved RNA, DNA polymerase fills the resulting gap and DNA ligase seals the nick [30], [31]. Previous studies suggest that the 3′ cut is made by FEN-1-like proteins [32] and that RNase HII nicking activity is promoted by binding to PCNA [33]. The importance of this pathway in repair of rNMPs incorporated by a DNA polymerase during replication has been emphasized by the observation that the lack of RNase HII in yeast strains expressing a mutant pol ε with relaxed sugar selectivity, leads to replicative stress and genome instability [34]. The main hallmark of this instability, deletion of 2–5 base pairs in short repetitive sequences, was also demonstrated in strains encoding wild-type pol ε and shown to require the endoribonuclease activity of Top1, a topoisomerase that relaxes supercoils by reversibly nicking duplex DNA [27]. The 2′-3′-cyclic phosphates formed after Top1-catalyzed cleavage between ribo- and deoxynucleotides, prohibit religation resulting in the generation of stable ssDNA breaks at the sites of incorporated rNMPs. Removal of all rNMPs is not a standard function of Top1, and it targets only some of the ribonucleotides in DNA/RNA hybrid when RNase H2 is defective [27].

Originally, it was hypothesized that the formation of deletions in cellular DNA in the absence of RNase HII occurs through a misalignment mechanism and involves mismatch repair (MMR) proteins, but it was subsequently shown to be independent of the status of the MMR machinery [35]. More recently it was revealed that the MMR system in both prokaryotes and eukaryotes competes with RNase H mechanisms to remove misincorporated ribonucleotides and restore DNA integrity when isolated rNMPs in chromosomal DNA also distort the Watson-Crick base-pairing [27].

The pathway of rNMP repair in prokaryotes is much less understood. Despite having multiple cellular functions, RNases HI and HII, encoded by the *rnhA* and *rnhB* genes respectively [29], are not essential for viability of bacteria, since the double mutants are viable, albeit temperature sensitive [36]. With respect to removal of rNMPs embedded in the genomic DNA, this means that other mechanisms can substitute for RNase H, or perhaps that prokaryotes can better tolerate DNA/RNA hybrid structures.

Taking advantage of the different capacities for ribonucleotide incorporation by pol V variants with substitutions at, or adjacent to the steric gate, we examined rNMP-processing pathways that cause phenotypic changes in strains expressing the pol V variants. We discovered that mutations in *rnhB* (encoding RNase HII), NER genes (*uvrA* and *uvrC*), and unexpectedly in *dinB* (encoding pol IV), play pivotal roles in modulating the extent of *umuC*_Y11A-dependent UV survival and mutagenesis. In addition, we show that in *recA730 lexA*(Def) Δ*dinB* strains lacking *rnhB*, *rnhA* helps to limit the extent of *umuC*_Y11A-dependent spontaneous mutagenesis imposed on the undamaged *E.coli* chromosome.

## Results

### Pol IV inhibits pol V–dependent incorporation of ribonucleotides during TLS *in vivo*

We have previously shown that the UV-resistance of *recA730 lexA*(Def) Δ*umuDC* cells expressing plasmid encoded *umuC*_Y11A is similar to that promoted by the vector plasmid alone [21]. This phenotype cannot be simply attributed to an inability to traverse the major UV-induced lesion because the highly purified pol V-Y11A enzyme bypassed the CPD efficiently *in vitro* [21]. Since UmuC_Y11A is characterized by low sugar discrimination fidelity [20], it seems plausible that the poor UV-survival of strains expressing the Y11A variant might be explained by large numbers of ribonucleotides incorporated during TLS that trigger repair pathways directed at rNMP removal. These pathways would excise pol V-dependent TLS tracts mimicking a pol V-deficient phenotype. To test this hypothesis, we measured the UV-sensitivity of strains expressing wild-type pol V and variants, but lacking an enzyme implicated in the repair of ribonucleotide-containing DNA. Based upon previous studies, the most logical choice was to assay UV-survival in strains lacking the RNase H proteins; RNase HI, the product of the *rnhA* gene [37] with the capacity to release RNA from RNA/DNA hybrids with multiple sequential rNMPs, and RNase HII encoded by the *rnhB* gene [38], which differs from the RNase HI by recognizing and cleaving a single ribonucleotide embedded in a DNA duplex. We therefore compared cell survival promoted by wild-type and mutant pol V variants in isogenic *recA730 lexA*(Def) Δ*umuDC* strains with Δ*rnhA* or Δ*rnhB* alleles alone, or in combination, after exposure to UV-light in a semi-quantitative “spot” assay. The plasmid-encoded pol V variants provide an excellent internal control for any effect of the RNase H proteins on cell survival, since the F10L mutant is essentially unable to incorporate ribonucleotides and should not exhibit any difference in the *rnh*+/− strains, whereas Y11A efficiently incorporates ribonucleotides and any rNMP-mediated repair would be most evident by comparison of the *rnh*+/− strains expressing this variant.

The first thing to note is that the Δ*rnhA* strain is more sensitive to UV-light than the isogenic *rnh* ^+^ or Δ*rnhB* strains (Figure S1), In this strain background, chromosomal duplication is dysregulated and unlike normal genome duplication which is initiated at *oriC,* is likely to be initiated at D-loops formed at *oriMs* and R-loops formed at multiple *oriKs* [39]. Presumably the added load of DNA damage to cells with highly irregular modes of replication contributes to the observed increased UV-sensitivity. In all strains analyzed, wild-type pol V conferred considerable UV-resistance (Figure S1).

As previously reported [21], in a *recA730 lexA*(Def) Δ*umuDC rnh* ^+^ background, *umuC*_Y11A confers minimal UV-resistance compared to either pGB2 vector, wild-type UmuC, Y11F, or F10L mutants (Figure S1A). In the Δ*rnhA* background, UV-survival of the Y11A variant was comparable to *umuC*_F10L and to the vector containing strain (Figure S1B). In contrast, while Y11A exhibited roughly the same overall UV-resistance as F10L in the Δ*rnhB* strain, it was considerably more UV-resistant than the vector containing strain (Figure S1C)

**Figure 1:**
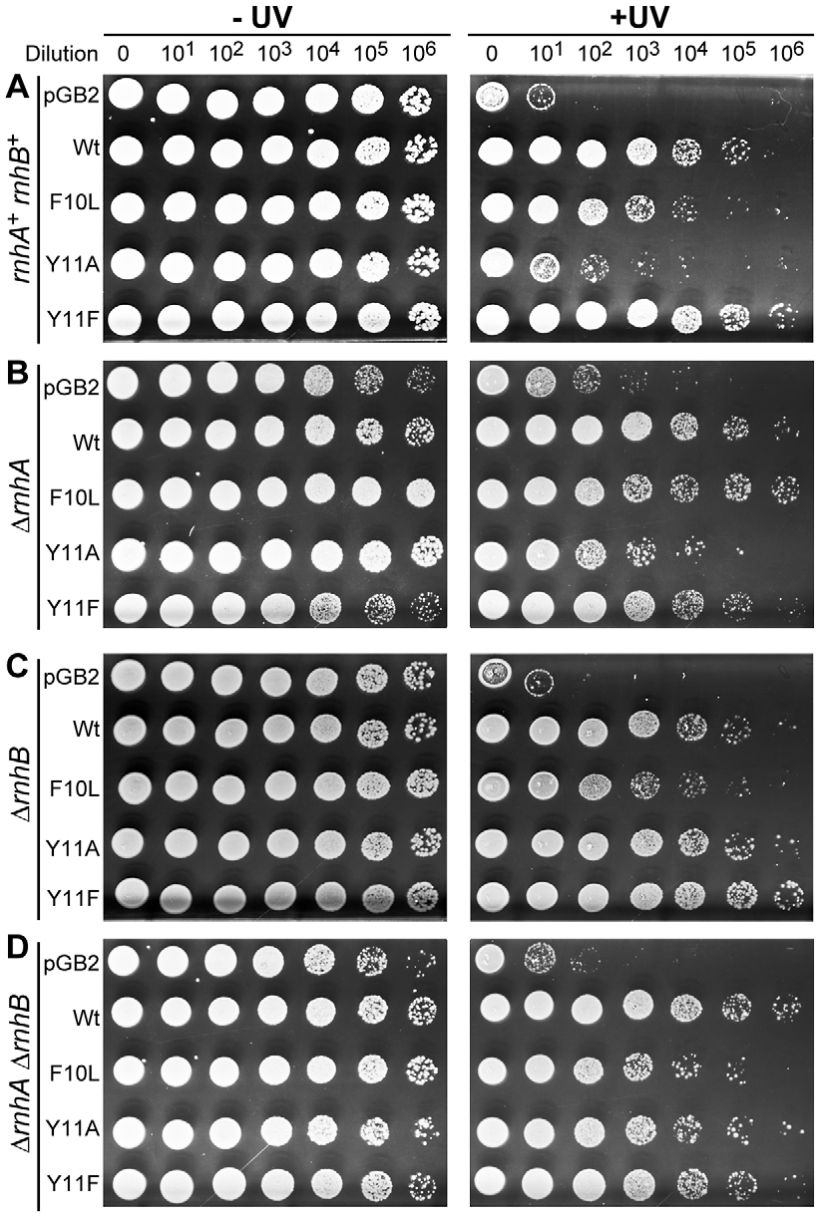
Effect of Δ*rnhA* and Δ*rnhB* on UV survival of *recA730 lexA*(Def) Δ*umuDC* Δ*dinB* strains expressing pol V variants. 10 µl of 10-fold serial dilutions of overnight cultures were spotted onto the surface of rectangular LB agar plates and exposed to 40 J/m^2^ 254 nM UV-light (panels A and C) and 20 J/m^2^ 254 nM UV-light (panels B and D). Both unirradiated (−) and UV-irradiated (+) plates were incubated overnight at 37°C. In each panel, UV survival is shown for the *recA730 lexA*(Def) Δ*umuDC* Δ*dinB* strains either harboring pGB2 vector, or expressing pol V variants. The main observation of these experiments is that the UV-resistance of cells expressing *umuC*_Y11A increase dramatically in strains lacking *rnhB*, whereas survival of cells equipped with wild-type pol V, *umuC*_F10L, or *umuC*_Y11F is largely unaffected by the status of *rnhB*.

We were concerned, however, that these phenotypes were much less pronounced than we had anticipated, especially given the properties of the Y11A mutant *in vitro* [20], [21]. The strains employed here carry the *lexA51*(Def) allele which has a frameshift mutation in the C-terminus of LexA [40], that leads to constitutive expression of all LexA-regulated genes [41], including all three TLS polymerases, pol II, pol IV and pol V. Under these conditions, pol IV is the most abundant DNA polymerase in *E.coli* with an intracellular concentration of roughly 2500 molecules per cell [42]. Even though pol IV has not previously been implicated in the TLS of UV-induced lesions, we considered the possibility that the highly abundant enzyme could nevertheless compete with pol V to limit its access to stalled replication forks. To test this hypothesis, we generated isogenic *recA730 lexA*(Def) Δ*umuDC* Δ*dinB* Δ*rnh*A, Δ*rnhB* or Δ*rnhA*-Δ*rnhB* mutant strains and re-analyzed the effects of defects in RNase H on UV-survival of cells expressing wild-type pol V and its variants (Figure 1). As observed previously (Figure S1), the Δ*rnhA* allele rendered the strains more UV-sensitive than the isogenic *rnh* ^+^ or Δ*rnhB* strains (Figure 1B and 1D, where cells were exposed to 20 J/m^2^ UV light compared to 40 J/m^2^ in Figure 1A and 1C). However, the Δ*rnhA* allele had no effect on the relative extent of UV-survival provided by the four pol V-expressing plasmids. In particular, the *umuC*_Y11A expressing plasmid conferred the least UV-resistance that was only marginally greater than the pGB2 vector containing strain.

A very different phenotype was observed in the Δ*rnhB* strain, where *umuC*_Y11A-dependent UV-survival was greatly enhanced (compare pGB2 and Y11A in Figure 1C). A similar enhancement of Y11A-dependent UV-survival was also observed in the more UV-sensitive Δ*rnhA* Δ*rnhB* double mutant strain (Figure 1D).

Overall, our data are consistent with the possibility that RNase H II activity actually promotes rNMP-dependent UV-induced cell killing. The fact that the increase in UV-resistance of Δ*rnhB* Y11A-expressing cells is much more dramatic in a Δ*dinB* background compared to the isogenic Δ*rnhB dinB* ^+^ strain implies that pol IV interferes with pol-V-catalyzed replication during TLS of CPDs. Such inhibition is surprising, since the prevailing models for TLS suggest that the two polymerases may cooperate to ensure efficient TLS [1]. Furthermore, It has been proposed that the more processive and catalytically efficient pol IV replaces pol V at the replication fork in order to protect the primer terminus from proofreading by the exonuclease-proficient enzymes [43].

To test whether the inhibitory effect on pol V is pol IV-specific, we generated isogenic *recA730 lexA*(Def) Δ*umuDC rnhB*+/− strains lacking pol II and determined UV-survival of cells expressing wild-type pol V and its variants (Figure S2). Deletion of pol II had very little effect on UV-resistance of the plasmid expressing strains. In general, in the *rnh* ^+^ strains the relative UV resistance of Y11A was comparable to that promoted by the vector, pGB2, and less than that conferred by the F10L plasmid. There was a modest increase in UV-resistance in the Δ*rnhB* strains (Figure S2), but this was comparable in the Δ*polB* and *polB* ^+^ strains and certainly much less evident than observed with the Δ*dinB*/*dinB* ^+^ Δ*rnhB* strains (c.f. Figure S1C and Figure 1C). Overall, our findings argue against a possible competition between pol II and pol V during the TLS of UV-induced lesions.

**Figure 2:**
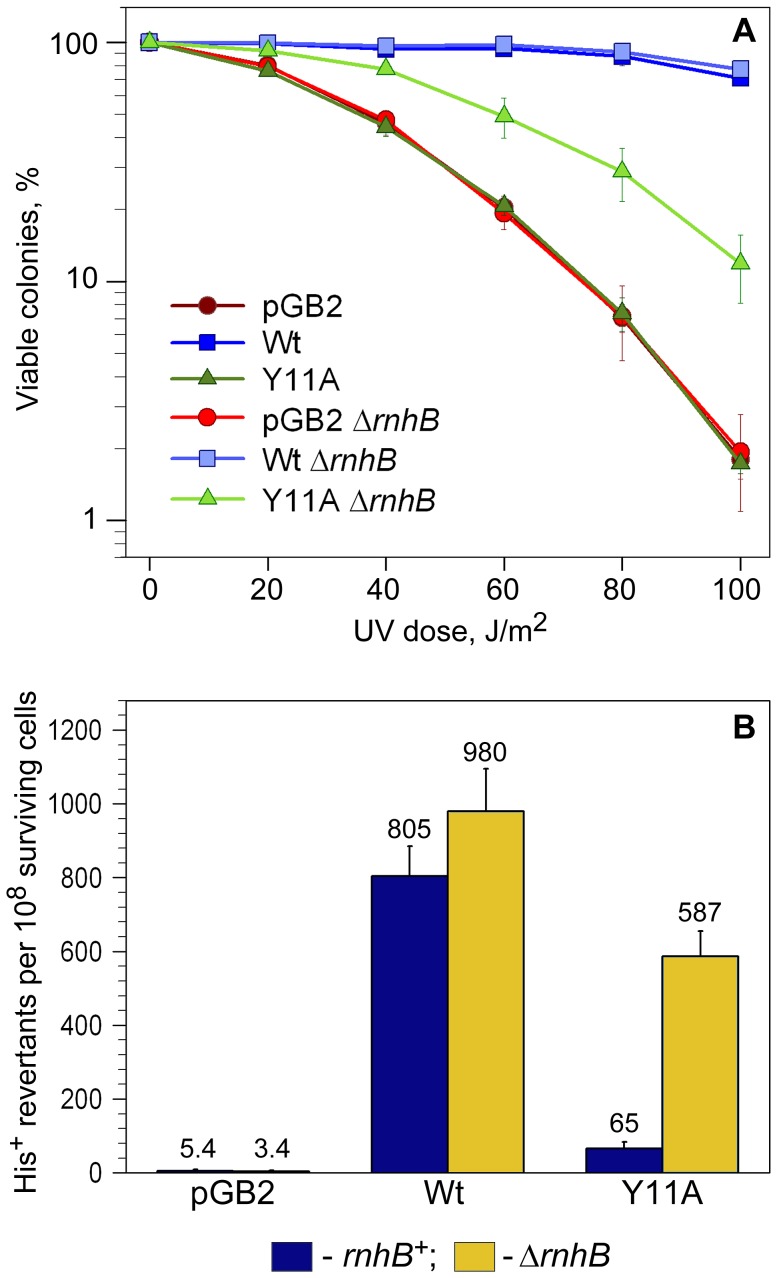
Quantitative UV survival and mutagenesis assays. A: Survival. Exponentially growing cells were exposed to various doses of UV-light and serial dilutions spread on LB plates containing spectinomycin. The number of viable colonies was determined after overnight incubation at 37°C. Error bars indicate the standard error of the mean. Consistent with the semi-quantitative UV-survival assay shown in Figure 1, UV-resistance of strains expressing Y11A_UmuC increased significantly in the Δ*rnhB* background, while there was no change in UV-survival of the strain harboring vector, pGB2, or expressing wild-type pol V in the *rnhB* ^+/−^ strains. B: Mutagenesis. UV-induced mutagenesis was determined by exposing exponentially growing cells to 20 J/m^2^ UV light. Cell viability was in the range of 85–90% survival for wild-type pol V and ∼60–70% for vector control, pGB2, and the Y11A mutant. The average number of His^+^ revertants per 10^8^ surviving cells ± standard error of the mean is indicated on the graph. The *rnhB* ^+^ strains are indicated by navy-colored bars, while Δ*rnhB* strains are indicated by the gold-colored bars. As observed, the UmuC_Y11A-expressing cells exhibited an ∼9-fold increase in UV mutagenesis compared to the *rnhB* ^+^ strain.

To characterize the effect of rNTP processing on UV-resistance and mutagenesis in strains expressing *umuC*_Y11A, we focused on the generally more UV-resistant *recA730 lexA*(Def) Δ*umuDC* Δ*dinB rnhB*+/− strains, rather than the more sensitive Δ*rnhA* derivatives, where the interpretation of any results might be complicated due to more complex phenotypes involving constitutive and induced stable DNA replication [39]. As shown in Figure 2A, the UV-survival curves of the Δ*dinB rnhB* ^+^ cells either lacking pol V, or expressing UmuC_Y11A are superimposable. Consistent with the semi-quantitative survival assay, the Δ*dinB* strain expressing UmuC_Y11A tolerates UV-damage much better in the absence of a functional RNase HII at all UV doses. In contrast, the absence of RNase HII has no effect on UV-resistance of strains either lacking pol V (pGB2), or expressing wild-type pol V.

Next, we compared the levels of UV-induced mutagenesis in the various strains by assaying reversion of the *hisG4* ochre allele (Figure 2B). While there was a slight reduction in the level of UV-mutagenesis promoted by wild-type pol V in the Δ*rnhB* strain compared to the *rnh* ^+^ strain, we observed an ∼9-fold increase in *umuC*_Y11A-dependent UV-induced mutagenesis in the Δ*rnhB* strain compared to the *rnhB* ^+^ strain (Figure 2B). Our observations therefore indicate that RNase HII plays a major role in preventing Y11A-dependent ribonucleotide-driven mutagenesis in *E.coli*.

The *rnhB* gene encoding RNase HII is located in a multi-gene operon and is immediately upstream of the *dnaE* gene encoding the catalytic α-subunit of pol III [44]. To eliminate the possibility that the observed phenotypes of the Δ*rnhB* allele on Y11A-dependent mutagenesis might be non-specific, due to effects on expression of *dnaE*, we determined the levels of the α-subunit in isogenic *dinB*+/− *rnhB*+/− strains (Figure S3). In both the *dinB*+/− cells, we observed an ∼25% reduction in the amount of α-subunit in the Δ*rnhB* strain compared to the *rnhB* ^+^ strain. The reduced levels of the α-subunit do not, however, explain the Y11A-dependent increase in UV-resistance and UV-mutagenesis in the Δ*rnhB* strains, since if reduced levels of the α-subunit allows greater access of pol V to a primer terminus, then we would have also expected to observe a significant increase in wild-type pol V UV-mutagenesis, when in fact, we actually observed a small decrease (Figure 2B)

### *In vitro* inhibition of pol V–dependent TLS by pol IV

**Figure 3:**
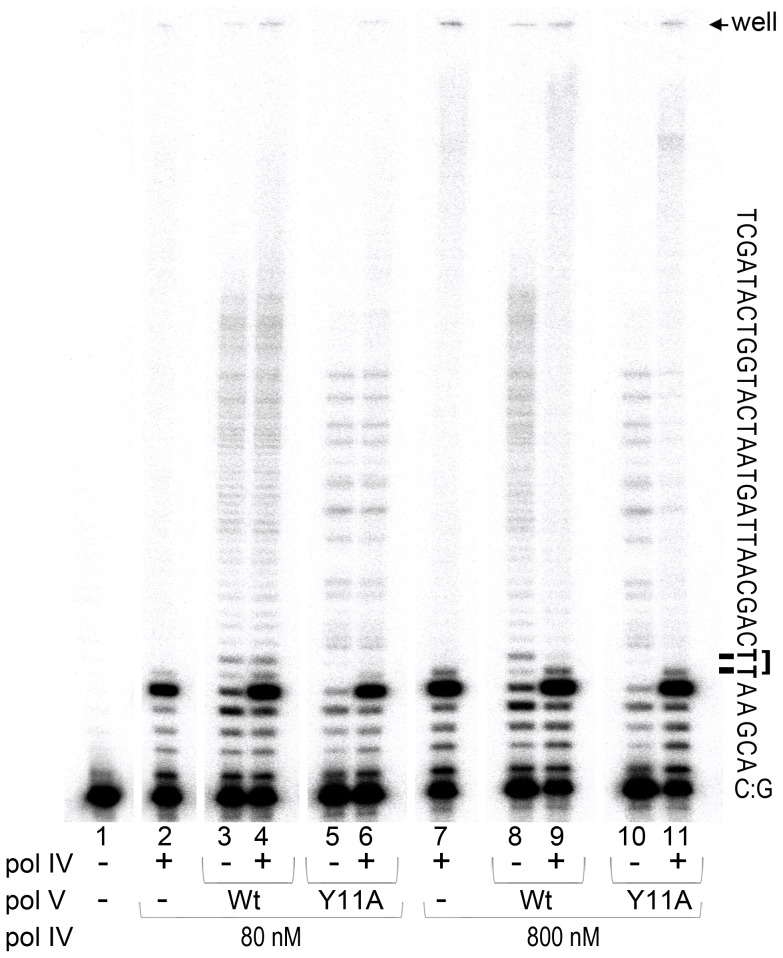
*In vitro* translesion synthesis past a TT-CPD lesion catalyzed by mixtures of pol IV and pol V. Translesion DNA synthesis was performed using a circular DNA template with a running-start primer with its 3′ end located 5 bases before the 3′T of the CPD. Primer extension reactions catalyzed by pol IV (80 nM, lane 1 or 800 nM, lane 6), wild-type pol V (80 nM, lanes 2 and 7), pol V (UmuC_Y11A) (60 nM, lanes 4 and 9), or a combination of pol IV (80 nM, lane 3 and 5 or 800 nM, lane 8 and 10) with either wild-type (80 nM, lanes 3 and 8) or polV (UmuC_Y11A) (60 nM, lanes 5 and 10) were performed for 30 sec as described in the Methods section. Part of the template sequence and position of the gel wells and a CPD lesion are indicated to the right of the gel panel. As clearly observed, when present in a 10-fold excess (similar to SOS induced conditions), pol IV inhibits TLS catalyzed by pol V.

Since pol IV appears to inhibit pol V-dependent TLS *in vivo*, we reconstituted TLS reactions *in vitro* using a circular vector with a unique T-T CPD [45], and a radiolabeled primer located five nucleotides 3′ from the CPD. To ensure maximal catalytic activity of both polymerases, the reaction conditions were optimized by including β-sliding processivity clamp, γ clamp-loading complex, and single-stranded DNA binding protein (SSB). The reactions also included a RecA nucleoprotein filament (RecA*), which has no noticeable effect on pol IV, but is required for pol V TLS *in vivo* [13]–[15] and *in vitro* [16], [17], [46]. The role of RecA* in pol V-catalyzed TLS is to transfer a molecule of RecA and ATP from its 3′-proximal tip to convert a barely active pol V to an activated UmuD′_2_C-RecA-ATP complex, termed pol V Mut [17], [46]. All reactions were carried out in a similar manner with some containing a single DNA polymerase, pol IV (Figure 3, lanes 2 and 7), wild-type pol V (lanes 3 and 8), or Y11A_UmuC pol V (lanes 5 and 10), while in other cases, pol V variants and pol IV were added simultaneously (lanes 4, 6, 9, and 11). DNA polymerases were used either at equi-molar concentrations (lanes 4 and 6), or with a 10-fold excess of pol IV over pol V (lanes 9 and 11).

As expected, pol IV by itself was unable to bypass a CPD adduct [18] even when used at elevated levels (Figure 3, lanes 2 and 7). The major reaction product observed was located immediately adjacent to the 3′ base of the CPD, with a very small band corresponding to nucleotide incorporation opposite the 3′T of CPD, as previously reported [18]. Although the overall primer extension efficiency of pol V was lower than that of pol IV (total primer extension by pol V ranged between 15 and 20%, while pol IV, depending on the concentration used, extended 35 or 85% of primers), the ability of pol V to replicate past the lesion was substantially greater. For example, ∼70% of primers extended by pol V to the −1 position (relative to the CPD) were further extended past the CPD. In contrast, and independent of the polymerase concentration used, only 3% of the primers bypassed the CPD when they were extended by pol IV. TLS catalyzed by wild-type pol V and Y11A pol V was similarly efficient and processive, even though the distribution pattern of products differed (Figure 3, lanes 3, 5, 8 and 10, see also [21]). When pol IV and pol V were used at ∼ equi-molar concentrations (lanes 2–6), the extent of lesion bypass catalyzed by wild-type pol V and pol V UmuC_Y11A was unaffected by the presence of pol IV since the amount of reaction products extended past the lesion remained the same. The apparent increase in the proportion of replication products that accumulated opposite the template A immediately 3′ to CPD (in lanes 4 and 6 compared to lanes 3 and 5), is compensated by the increased proportion of elongated primers suggesting that pol V was unable to replace pol IV at the lesion site. When pol IV was present at ∼10-fold excess, which is roughly equivalent to the *in vivo* cellular ratio when maximally expressed during SOS-induction [42], [47] (lanes 7–11), significant inhibition of pol V-dependent TLS was observed (compare lanes 9 and 11 with lanes 8 and 10). In addition, the general distribution pattern of reaction products was similar to that observed in the reaction containing only pol IV (compare lanes 9 and 11 to lane 7, and the amount of primer elongated past the lesion expressed as a percent of primers elongated to the −1 position, was reduced to 3%, which is equivalent to the results observed in reactions in the presence of pol IV alone). The data suggests that under certain SOS-inducing conditions, pol IV may bind to the 3′-primer terminus of nascent DNA strand and thereby prevent access of pol V to the replicating fork thus serving as a cellular “competitive” inhibitor of pol V-dependent TLS at DNA lesions that pol IV itself is unable to bypass.

### Effect of nucleotide excision repair on UV resistance of cells actively repairing misincorporated rNMPs

**Figure 4:**
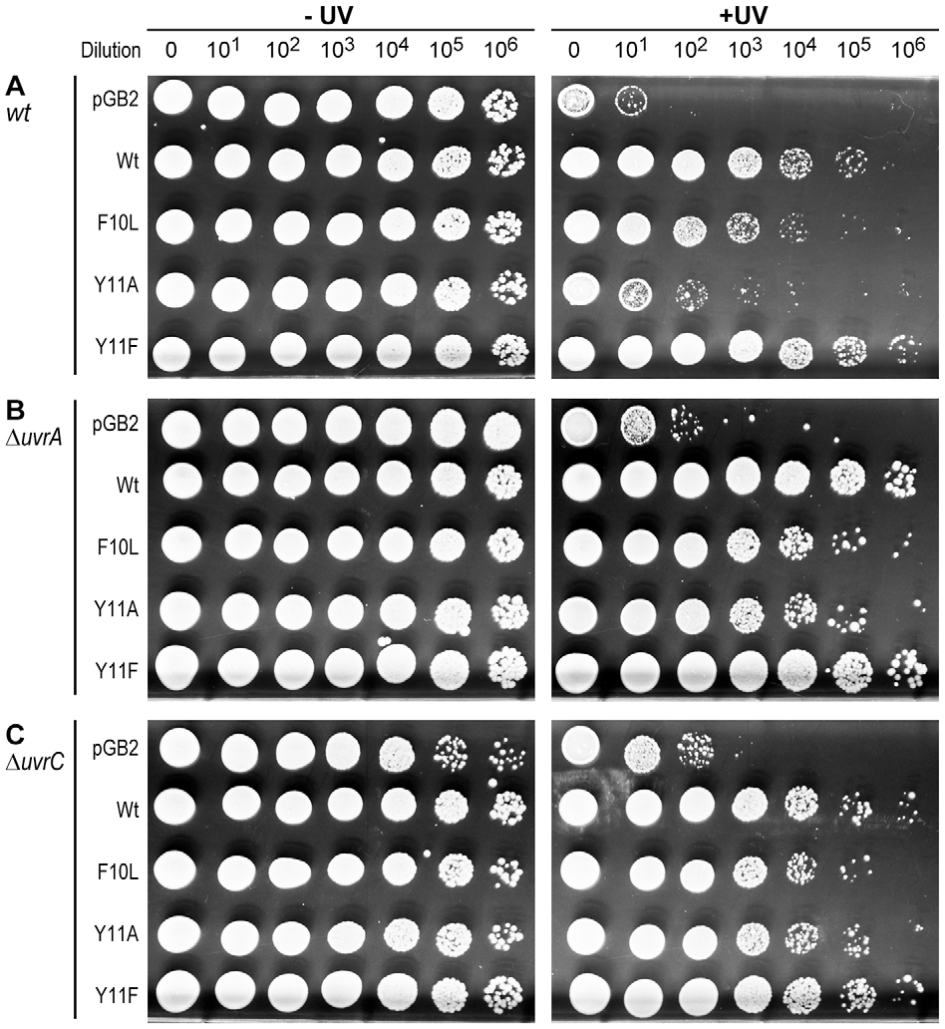
Role of NER in strains expressing pol V variants. 10 µl of 10-fold serial dilutions of overnight cultures were spotted onto the surface of rectangular LB agar plates and exposed to 40 J/m^2^ 254 nM UV-light (Panel A), or 1 J/m^2^ 254 nM UV-light (Panels B and C). Both unirradiated (−) and UV-irradiated (+) plates were incubated overnight at 37°C. In each panel, UV survival is shown for the *recA730 lexA*(Def) Δ*umuDC* Δ*dinB* strains either harboring pGB2 vector, or expressing pol V variants. Panel A (*uvr* ^+^ strain) is reproduced from Figure 1A for direct comparison to the isogenic Δ*uvrA* (Panel B) and Δ*uvrC* (panel C) strains. The main observation of these experiments is that while the *uvr* ^−^ strains are considerably more UV-sensitive than the isogenic *uvr* ^+^ strain, the relative sensitivity of the strains expressing pol V variants changes in the *uvr* ^−^ background, with UmuC_Y11A promoting an increase in UV-survival to a similar extent as wild-type pol V.

Our previous studies [21], and those described above (Figure 1 and Figure 2), indicate that in a strain actively repairing errantly-incorporated ribonucleotides, expression of *umuC*_Y11A confers minimal UV-resistance compared to wild-type pol V, or other pol V variants (*umuC*_F10L or *umuC*_Y11F). This phenotype, can, in part, be explained by the fact that the abundant ribonucleotides target the pol V-generated TLS tract for repair. As shown above, this process is initiated by RNase HII, which nicks the DNA backbone immediately 5′ of the misincorporated ribonucleotide, but the ribonucleotide must subsequently be physically replaced using other repair enzymes/polymerases with a limited ability to traverse UV-induced DNA lesions. To identify proteins involved in ribonucleotide removal, we constructed a series of isogenic *recA730 lexA*(Def) Δ*umuDC* Δ*dinB* strains with individual deletions of various DNA repair genes (unpublished data) and determined whether or not such an inactivation would lead to an increase in *umuC*_Y11A-specific UV-resistance, in a similar manner to that observed with defects in *rnhB* (Figure 1D and Figure 2). The control, or pol V-encoding plasmids were introduced into these repair deficient strains, and their sensitivity to UV-light was assayed in the semi-quantitative UV survival assay. In most cases, the ability of the particular pol V plasmid to confer UV resistance was the same as shown in Figure 4A, i.e., the relative UV-sensitivity of each plasmid-expressing strain remained the same. Wild-type pol V was similar to Y11F, and both were better than F10L at promoting UV-survival, while Y11A conferred minimal UV-resistance (unpublished data). However, a markedly different pattern of survival emerged in strains defective for nucleotide excision repair (NER), such as *uvrA*, which plays a critical role in damage recognition (Figure 4B), or *uvrC*, an endonuclease which cleaves the phosphodiester bond 3′ of the lesion, (Figure 4C). As expected because of their deficiency in NER, the Δ*uvrA* and Δ*uvrC* strains were much more sensitive to UV-light than the isogenic parental strains. As a consequence, while cells shown in Figure 4A were exposed to 40 J/m^2^ UV-light, the NER deficient cells shown in Figure 4B and 4C were only exposed to 1 J/m^2^ UV. However, the key observation is that in the NER-deficient strains, *umuC*_Y11A conferred considerable UV-resistance that was roughly similar to that observed for wild-type pol V and the *umuC*_Y11F variant (Figure 4B and 4C). Thus, the status of the NER machinery plays a critical role in the survival of cells exposed to DNA damage whilst concomitantly incorporating high levels of ribonucleotides.

### Effect of RNase H on the levels of spontaneous mutagenesis in strains expressing pol V and variants

**Figure 5:**
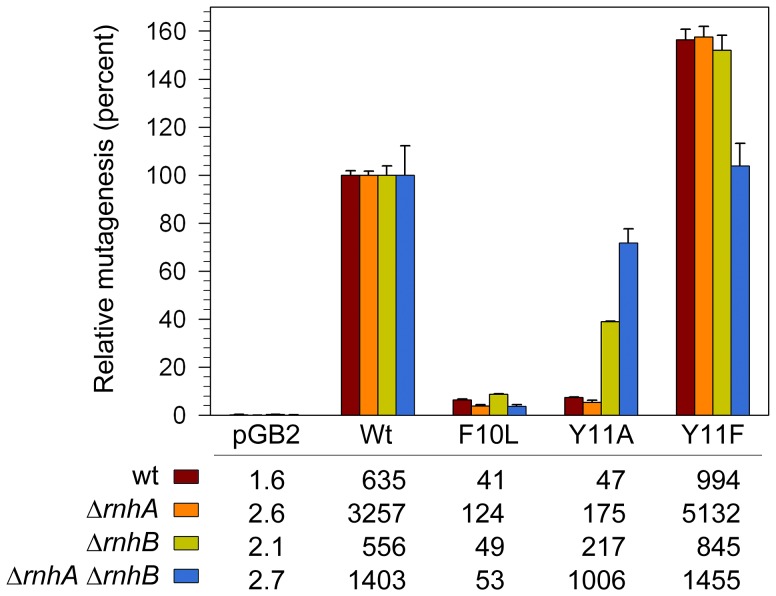
Effect of Δ*rnhA* and Δ*rnhB* on spontaneous mutagenesis in *recA730 lexA*(Def) Δ*umuDC* Δ*dinB* strains expressing pol V variants. Spontaneous mutagenesis was measured by assaying reversion of the *hisG4* ochre allele (leading to *histidine* prototophy) as described in *Materials and Methods*. The average number of His^+^ revertants per plate ± standard error of the mean is indicated in the table. Since the extent of mutagenesis promoted by wild-type pol V differed in the various strains, we have expressed the level of mutagenesis promoted by the variants as a percentage of wild-type mutagenesis. As clearly observed, *umuC*_Y11A-dependent mutagenesis increased in the Δ*rnhB* strain and was further elevated in the Δ*rnhA* Δ*rnhB* double mutant. In contrast, *umuC*_F10L gave consistently low levels of mutagenesis in all strains, and *umuC*_Y11F higher than wild-type levels in all strains.

In addition to facilitating TLS, when expressed in a *recA730 lexA*(Def) background, pol V promotes high levels of spontaneous mutagenesis [15]. This mutagenesis is not the result of TLS of “cryptic” DNA lesions, but rather the ability of pol V to compete with *E.coli*'s other DNA polymerases and gain access to undamaged genomic DNA where its low-fidelity synthesis is manifested as mutagenic events on the *E.coli* chromosome [48]. We have shown that despite its low sugar and base-substitution fidelity *in vitro*, when expressed in a *recA730 lexA*(Def) Δ*umuDC* strain, *umuC*_Y11A promotes low levels of spontaneous mutagenesis [20]. One obvious explanation, based upon our observations above, is that mutagenesis is limited via the actions of *rnhB*. However, the strain used in the earlier study also expresses pol IV and while it is believed that pol V and pol IV work together to promote spontaneous mutagenesis [43], we could not exclude the possibility that in a similar manner to its negative effect on the TLS of CPDs, pol IV might actually block access of the error-prone pol V_Y11A polymerase to undamaged chromosomal DNA. To test this hypothesis, we assayed spontaneous mutagenesis in isogenic *recA730 lexA*(Def) Δ*umuDC* Δ*dinB rnh* ^+/−^ strains (Figure 5). Expression of wild-type pol V in the *rnhB* ^+^ cells resulted in a substantial increase in the number of spontaneously arising His^+^ revertants compared to the same strain lacking pol V. In the isogenic Δ*rnhA* strain, there was a considerable (3–5-fold) increase in the number of revertants promoted by pol V and variants, presumably because the increased number of R-loops in the Δ*rnhA* strain [39] help to hyperactivate the RecA730 protein [49] for its role in pol V-dependent mutagenesis [17]. While there was a 3.7-fold increase in the absolute number of Y11A-dependent mutations in the Δ*rnhA* strain compared to *the rnh* ^+^ strain, when expressed as a percentage of wild-type pol V-dependent mutagenesis, *umuC*_Y11A mutagenesis actually decreased from 7 to 5% of the wild-type levels (Figure 5). In contrast, in the isogenic Δ*rnhB* strain, the number of *umuC*_Y11A-dependent revertants increased approximately 4.6-fold compared to the *rnhB* ^+^ strain and reached ∼40% of the level of mutagenesis observed with wild-type pol V (Figure 5).

Our studies therefore show that RNase HII clearly participates in a repair pathway that reduces the accumulation of rNMPs, as well as incorrect dNMPs incorporated into undamaged and damaged DNA by UmuC_Y11A. Based upon its *in vitro* properties [21], we expected pol V *umuC*_Y11A to be as mutagenic, if not more so, than the wild-type pol V, but even in the Δ*rnhB* strain, Y11A-dependent mutagenesis was less than half of that observed with wild-type pol V (Figure 5), suggesting that perhaps additional repair pathways act to reduce the mutagenic consequences of rNMPs incorporated by the highly error-prone *umuC*_Y11A. Indeed, in the isogenic Δ*rnhA* Δ*rnhB* strain *umuC*_Y11A spontaneous mutagenesis increased significantly to ∼72% of the level observed with wild-type pol V (Figure 5). Thus, although Rnase HI alone does not appear to participate in the removal of ribonucleotides incorporated by *umuC*_Y11A, in the absence of Rnase HII, where there is likely to be a significant accumulation of ribonucleotides into DNA, Rnase HI helps reduce the mutagenic burden of errant ribonucleotide incorporation into the *E.coli* genome.

## Discussion

In order to maintain its genomic integrity a cell must protect its DNA from constant assaults coming from different sources. Among these is the attempt to replace the sugar moiety of a nucleotide, which appears to be one of the most persistent potential sources of “damage”. Incorporation of rNMP into the DNA backbone most frequently occurs during DNA replication and repair due to mistakes made by DNA polymerases. Seemingly a harmless event, assuming that the base of the ribonucleotide being incorporated is a correct Watson-Crick pair, it nevertheless can threaten the cell's well-being because the presence of rNMP with a reactive 2′ hydroxyl on the ribose ring makes the DNA strand more susceptible to spontaneous or enzymatic cleavage. It can also lead to a *B-* to *A*-form conformational DNA transition and disrupt interactions of DNA-binding proteins thereby compromising various DNA processing pathways. *E.coli* pol V appears to be one of the least discriminate DNA polymerases. The sugar selectivity of pol V can be significantly improved by an F10L substitution in the catalytic subunit UmuC and vice versa, a Y11A substitution in UmuC significantly reduces the ability of pol V to select a nucleotide with the correct sugar [20].

To prevent the deleterious effects of ribonucleotides incorporated into DNA, *E.coli* is equipped with enzymes capable of hydrolyzing the phosphodiester bond between ribo- and deoxyribonucleotides, thereby triggering repair pathways leading to removal of rNMPs. In the present study, we show that ribonucleotide-specific endonuclease RNase HII plays an important role in the correction of mistakes made by error-prone pol V. The basic mechanism of ribonucleotide repair appears to be evolutionary conserved as Nick McElhinny *et al*., recently reported that RNase H2-dependent repair is necessary for the prevention of replicative stress and genome instability in yeast strains expressing a pol ε variant with compromised sugar selectivity [23]. However, in contrast to other studies demonstrating that deletion of RNase H2 increases spontaneous mutagenesis in yeast strains with wild-type DNA polymerases [25], [50], [51], no increase in spontaneous or UV-induced mutagenesis was observed upon in a Δ*rnhB* strain expressing wild-type pol V (Figure 2B and Figure 5). Nevertheless, these data do not imply that RNase HII is not important for correction of pol V-dependent mistakes, but rather suggest that sugar selectivity of the polymerase must be significantly reduced for endonuclease function to be readily detectable. Indeed, the lack of RNase HII caused a significant increase in mutagenesis in strains expressing UmuC_Y11A for which ribonucleotide processing is most important. Therefore, the pathway initiated by RNase HII not only leads to removal of nucleotides with an incorrect sugar, but also to the repair of base substitutions, explaining the low mutability of the *rnhB^+^* strain expressing highly error-prone UmuC_Y11A.

While we observed a minimal effect of Δ*rnhA* alone on the level of *umuC*_Y11A-dependent spontaneous mutability, there was a dramatic increase in spontaneous mutagenesis in combination with the Δ*rnhB* allele (Figure 5). Presumably this is due to the accumulation of ribonucleotides in the absence of Rnase HII, and the propensity of the Y11A variant to catalyze synthesis of polynucleotide chains containing multiple sequential rNMPs [20].

It should also be noted that the role of RNase HII-initiated repair in determining UV-sensitivity of Y11A-expressing cells was most pronounced in Δ*dinB* strains. We assume that in cells expressing *dinB* (Pol IV), there is competition for a primer-terminus between pol IV and pol V that limits the extent of the pol V-dependent TLS tract, which in the case of UmuC_Y11A will concomitantly reduce the number of incorporated rNMPs into the genome, and the need for RNase HII-mediated repair (Figure S1C). In a similar vein, since the number of rNMPs incorporated by the wild-type pol V, UmuC_Y11F, and especially UmuC_F10L pol V, is significantly lower than that of UmuC_Y11A, the effect of RNase HII and pol IV on UV-resistance is negligible in the strains expressing these polymerases (Figure 1).

**Figure 6:**
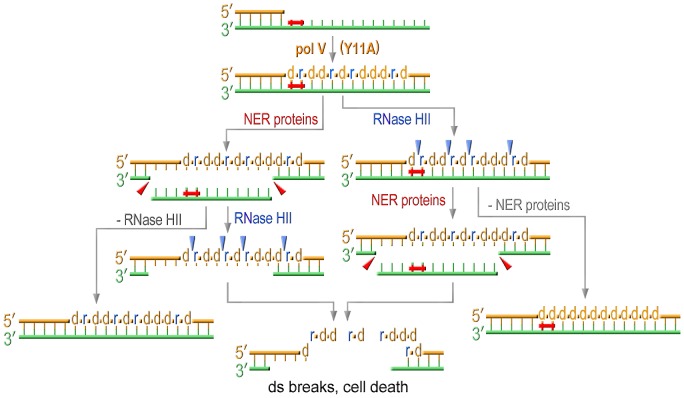
Model for the effect of RNase HII and NER proteins on UV sensitivity of strains proficient for ribonucleotide incorporation. Translesion replication catalyzed by Y11A mutant produces a TLS tract containing multiple ribonucleotides. NER excises UV-induced lesions and produces gaps on the template strand. RNase HII initiating removal of multiple rNMPs incorporated during TLS produces nicks on the daughter strand. The concerted action of both these repair pathways results in formation of persistent double strand breaks ultimately leading to cell death. Inactivation of either repair pathway selectively improves UV-resistance of cells expressing UmuC_Y11A.

RNase HII-initiated rNMP repair, which at the same time leads to the correction of base substitutions, readily explains the low mutability of strains expressing a pol V variant with an impaired steric gate. In the current study, we found that the low UV-resistance, which is somewhat unexpected for cells equipped with an efficient TLS polymerase such as UmuC_Y11A [20], is also connected to ribonucleotide incorporation and repair. For example, we show that the sensitivity to UV-light in cells expressing *umuC*_Y11A is reduced by RNase HII to the level detected in the strain completely lacking pol V. The observation that removal of rNMPs actually diminishes cells viability implies that unlike yeast, in which unrepaired rNMPs lead to genome instability, bacterial cells can tolerate the presence of ribonucleotides in genomic DNA quite well. This assumption is supported by the findings that all the strains defective in rNMPs repair have similar colony size and growth rates independent of the sugar discrimination properties of a pol V variant. However, even assuming that *E.coli* is able to tolerate the presence of ribonucleotides in its genome, it nevertheless seems counterintuitive that activation of a pathway directed at the removal of rNMPs would reduce cell viability after UV-treatment. In order to explain these observations, we propose the following model: simultaneous attempts of the NER machinery to repair UV-induced lesions on the parental template strand and of the RNase HII-initiated pathway to remove numerous rNMPs from the TLS-tract in the nascent strand, lead to the formation of multiple and persistent DNA double-strand breaks that cause cell death (Figure 6). Pulse Field Gel Electrophoresis (PFGE) and bacterial COMET assays are currently underway to test this hypothesis. When either of these repair pathways is inactivated, UmuC_Y11A confers significant UV resistance (Figure 1D, Figure 2A, and Figure 4) presumably because of its ability to facilitate TLS and in spite of the fact that ribonucleotides are concomitantly incorporated into the *E.coli* genome. However, the increased survival comes at the steep cost of increased cellular mutagenesis (Figure 2B, Figure 5).

In summary, *E.coli* utilizes a variety of mechanisms to minimize pol V-dependent ribonucleotide incorporation into its genome. The first line of defense is a competition between pol IV and pol V during TLS that limits the access of the errant pol V to a stalled primer terminus. In the absence of pol IV, pol V can incorporate ribonucleotides, but these are rapidly removed from the genome in an RNase HII-initiated repair pathway. Concurrent with rNTP removal, NER of the UV-lesion results in double-strand breaks leading to cell death. The Rnase HII-mediated repair pathway minimizes both UV-induced and spontaneous mutagenesis to the bacterial chromosome and in its absence, and in the presence of a mutant pol V with a propensity to incorporate polyribonucleotides (UmuC_Y11A), RNase HI serves as a backup to RNase HII to limit the mutagenic consequences of excessive ribonucleotide accumulation into the *E.coli* genome.

## Materials and Methods

### Bacterial strains

Most of the *E. coli* K-12 strains used in this study are derivatives of RW584 (full genotype: *recA730 lexA51*(Def) Δ*umuDC596*::*ermGT thr-1 araD139* Δ(*gpt-proA*)*62 lacY1 tsx-33 glnV44 galK2 hisG4 rpsL31 xyl-5 mtl-1 argE3 thi-1 sulA211* [52]. All derivatives were made by standard methods of P1 transduction using P1*vir* [53] (Table 1). The various alleles were selected by conferring resistance to spectinomycin (20 µg/ml), zeocin (25 µg/ml), chloramphenicol (20 µg/ml) and kanamycin (50 µg/ml) respectively and subsequently confirmed by PCR [54]–[56].

**Table 1:** *E. coli* strains used in this study.

Strain	Relevant Genotype	Source or Reference
RW584^a^	*recA730 lexA51*(Def) Δ*(umuDC)596*::*ermGT*	[52]
STL1366	Δ*polB*::ΩSpec	[61]
AR30	Δ*dinB61*::*ble*	[62]
MIC1024	Δ*rnhA339*::*cat*	[55]
JW0178	Δ*rnhB782*::Kan	*E.coli* Genetic Stock Center
JW4019	Δ*uvrA753*::Kan	*E.coli* Genetic Stock Center
JW1898	Δ*uvrC759*::Kan	*E.coli* Genetic Stock Center
RW820^a^	*recA730 lexA51*(Def) Δ*(umuDC)596*::*ermGT* Δ*rnhA339*::*cat*	RW584×P1 MIC1024
RW822^a^	*recA730 lexA51*(Def) Δ*(umuDC)596*::*ermGT* Δ*rnhB782*::Kan	RW584×P1. JW0178
RW702^a^	*recA730 lexA51*(Def) Δ*(umuDC)596*::*ermGT* Δ*polB*::ΩSpc	RW584×P1. STL1366
RW934^a^	*recA730 lexA51*(Def) Δ*(umuDC)596*::*ermGT* Δ*polB*::ΩSpc Δ*rnhB782*::Kan	RW702×P1. JW0178
RW698^a^	*recA730 lexA51*(Def) Δ*(umuDC)596*::*ermGT* Δ*dinB61*::*ble*	RW584×P1. AR30
RW1044^a^	*recA730 lexA51*(Def) Δ*(umuDC)596*::*ermGT* Δ*dinB61*::*ble* Δ*rnhA339*::*cat*	RW698×P1. MIC1024
RW838^a^	*recA730 lexA51*(Def) Δ*(umuDC)596*::*ermGT* Δ*dinB61*::*ble* Δ*rnhB782*::Kan	RW698×P1. JW0178
RW1092^a^	*recA730 lexA51*(Def) Δ*(umuDC)596*::*ermGT* Δ*dinB61*::*ble* Δ*rnhA339*::*cat* Δ*rnhB782*::Kan	RW1044×P1. JW0178
RW902^a^	*recA730 lexA51*(Def) Δ*(umuDC)596*::*ermGT* Δ*dinB61*::*ble* Δ*uvrA753*::Kan	RW698×P1. JW4019
RW906^a^	*recA730 lexA51*(Def) Δ*(umuDC)596*::*ermGT* Δ*dinB61*::*ble* Δ*uvrC759*::Kan	RW698×P1. JW1898

a : *thr-1 araD139* Δ*(gpt-proA)62 lacY1 tsx-33 glnV44 galK2 hisG4 rpsL31 xyl-5 mtl-1 argE3 thi-1 sulA211*.

### Plasmids

The low-copy-number plasmids used for expression of UmuC variants are derived from pGB2 [57]. Spectinomycin resistant plasmids pRW134, pJM964, pJM963, and pJM952 encode *E.coli* UmuD′ along with wild-type UmuC or F10L, Y11A, and Y11F variants, respectively and the proteins are expressed from the native *umu* promoter [20]. Ampicillin resistant derivatives were generated by replacing the *Bsp*HI-*Bsp*HI vector fragment encoding resistance to spectinomycin with a *Bsp*HI-*Bsp*HI fragment from pET22b+ encoding resistance to ampicillin. Bacteria harboring plasmids were grown in LB media containing appropriate antibiotics (50 µg/ml spectinomycin, or 100 µg/ml ampicillin).

### Semi-quantitative UV survival assays

Cells were grown overnight at 37°C in Luria–Bertani (LB) plus spectinomycin. The next morning, the cultures were sequentially diluted 10-fold in eppendorf tubes containing SM buffer [58]. 10 µl of each serial dilution was then spotted on the surface of a 12×8 cm rectangular LB agar plate (Nunc, ThermoFisher). The plates were irradiated with UV light (254 nm) and incubated overnight at 37°C. Images of the irradiated plates/cultures were captured with a FluorChem HD2 imaging system (Alpha Innotec).

### Quantitative spontaneous mutagenesis, UV-induced mutagenesis, and UV survival assays

Cells transformed with the vector plasmid, pGB2, or one of the low-copy number plasmids expressing wild-type pol V or UmuC variants were grown overnight at 37°C in LB media plus spectinomycin. The next day, cultures were diluted 100-fold into 10 ml fresh media and grown at 37°C until they reach an OD_600_ of 0.1 (roughly 3 hrs). Cells were centrifuged and resuspended in an equal volume of SM buffer [58] and transferred to a petri dish. Aliquots were removed and saved as the unirradiated control for the experiments. The culture was irradiated at a UV fluence of ∼2 J/m^2^ per second and aliquots removed at 20 J/m^2^ increments. At least three independent cultures were assayed for each strain and all experiments were performed under yellow light to avoid unwanted photoreactivation.

For UV survival assays, appropriate serial dilutions (based upon trial assays) were plated on LB agar plates containing spectinomycin and incubated overnight at 37°C. The surviving fraction was determined by dividing the number of viable cells exposed to UV by the number of viable cells in the unirradiated culture. Error bars represent the standard error of the mean (SEM).

To determine the number of spontaneously arising histidine mutants on the plate, as well as UV-induced mutants, the unirradiated cell culture was seeded on the Davis and Mingioli minimal agar plates [59] plus glucose (0.4% wt/vol); agar (1.0% wt/vol); proline, threonine, valine, leucine, and isoleucine (all at 100 µg/ml); thiamine (0.25 µg/ml); and either no histidine, or histidine (1 µg/ml). On the plates containing no histidine, only pre-existing His^+^ mutants grew to form colonies. However, on the plates containing 1 µg/ml histidine, 100–200 His^−^ cells are able to grow on the limiting amount of histidine, so that a viable cell count can be obtained under the exact same conditions where His^+^ mutant arise. When ∼4×10^7^ bacteria were seeded, they grew to form a lawn, concomitantly exhausting the low level of histidine. Spontaneously arising His^+^ mutants grew up through the lawn and were counted after 4 days incubation at 37°C.

To determine the extent of UV-induced mutagenesis, cells that had been irradiated with 20 J/m^2^ UV were used for analysis. This UV dose was chosen since even the UV-sensitive strains exhibited minimal cell killing at this exposure. These conditions therefore provide a window to observe UV-induced mutagenesis without the complications associated with differential levels of cell killing in the various strains. The UV-induced mutation frequencies were calculated as previously described [60]. This equation not only takes into account the number of mutants spontaneously arising on the low histidine plates, but also any effect of reduced cell viability on the number of pre-existing His^+^ mutants in the culture. The data reported in Figure 4 represent the average number of His^+^ mutants from 3 separate experiments (± standard error of the mean [SEM]).

### Western blots

Cells were grown overnight at 37°C in LB plus appropriate antibiotics. The next morning, cultures were diluted 1∶100 in fresh LB, plus antibiotics and grown with aeration at 37°C until they reached an OD_600_ of ∼0.5. Cultures were harvested by centrifugation, resuspended in 1× SDS sample buffer (50 mM Tris-HCl [pH 6.8], 10% glycerol, 2.3% sodium dodecyl sulfate [SDS], 0.1% bromophenol blue, 10 mM dithiothreitol), and immediately frozen in dry ice. Cells were lysed by multiple freeze-thaw cycles and boiled for 5 mins at 95–100°C. Extracts were immediately applied to a 15% SDS-PAGE gel. After separation, proteins were transferred to an Immobilon-P membrane (Millipore) using standard Western blot protocols. The membrane was incubated overnight with a 1∶1000 dilution of mouse monoclonal antibodies raised against the α-subunit of pol III (kindly provided by Charles McHenry, University of Colorado). The membrane was then incubated with secondary anti-mouse alkaline phosphatase conjugated antibodies and visualized using the CSPD-Western light assay (Applied Biosystems). Pictures were captured on a FluorChem HD2 imaging system (Alpha Innotec).

### *In vitro* replication assays

Wild-type pol V, the UmuC_Y11A variant and pol IV, β-clamp and γ-complex were purified as previously described [46]. pSOcpd plasmid, containing a unique CPD adduct, was also constructed as previously described [45]. All oligonucleotides were synthesized by Lofstrand Laboratories (Gaithersburg, MD) and gel purified prior to use. 5′-^32^P labeled M13-TT (5′ – GAT-CGA-TGG-TAC-GGA-CG) primer was annealed to pSOcpd ssDNA templates at a 1.5∶1 molar ratio by heating in an annealing buffer (50 mM Tris-HCl (pH 8), 5 mM MgCl_2_, 50 µg/ml BSA, 1.42 mM 2-mercaptoethanol) for 10 min at 100°C followed by slow cooling to room temperature. 4 mM RecA (New England Biolabs, Ipswich, MA) was incubated with 0.25 µM 48-mer single-stranded oligonucleotide in the presence of 1 mM adenosine 5′[γ-thio]triphosphate (ATPγS, Biolog Life Science Institute, Bremen, Germany) in the 1× reaction buffer [20 mM Tris-HCl pH 7.5, 8 mM MgCl_2_, 8 mM DTT, 80 µg/ml BSA, 4% glycerol] at 37°C for 5 min to form RecA nucleoprotein filament on ssDNA (RecA*). Reaction mixture containing 1 mM ATP, 50 µM dNTPs, 2 nM DNA templates, 100 nM SSB (Epicentre Biotechnologies, Madison WI), 50 nM β clamp, and 5 nM γ complex in the 1× reaction buffer was preincubated for 3 min at 37°C. Purified pol V variants (80 nM) were first combined with RecA*(0.25 µM) to form pol V Mut and then added to the reaction mixture. When indicated, Pol V was either substituted, or mixed with indicated amounts of purified pol IV. Reactions were incubated at 37°C for 20 mins and terminated by adding 10 ml of 2× loading buffer [97% formamide, 10 mM EDTA, 0.1% xylene cyanol, 0.1% bromophenol blue]. The products were heat-denatured and resolved by denaturing PAGE (8 M urea, 15% acrylamide), followed by visualization on a Fuji image analyzer FLA-5100.

## Supporting Information

Figure S1 UV-survival of *recA730 lexA*(Def) Δ*umuDC* strains expressing pol V variants. Ten microliters of 10-fold serial dilutions of overnight cultures were spotted onto the surface of rectangular LB agar plates and exposed to 40 J/m^2^ (panels A and C) and 10 J/m^2^ (panel B) 254 nM UV-light. Both unirradiated (−) and UV-irradiated (+) plates were incubated overnight at 37°C. In each panel, UV survival is shown for the *recA730 lexA*(Def) Δ*umuDC* strains either harboring pGB2 vector, or expressing pol V variants.
(PDF)

Figure S2 UV-survival of *recA730 lexA*(Def) Δ*umuDC polB* ^+^/Δ*polB* strains expressing pol V variants. Panel A and C, *rnhB* ^+^; Panel B and D, Δ*rnhB*. Ten microliters of 10-fold serial dilutions of overnight cultures were spotted onto the surface of rectangular LB agar plates and exposed to 40 J/m^2^ 254 nM UV-light. Both unirradiated (−) and UV-irradiated (+) plates were incubated overnight at 37°C. In each panel, UV survival is shown for the *recA730 lexA*(Def) Δ*umuDC* strains either harboring pGB2 vector, or expressing pol V variants.
(PDF)

Figure S3 Western blot of the α-subunit of pol III holoenzyme in *rnh* ^+^ and Δ*rnhB* strains. The α-subunit of pol III holoenzyme was detected in whole cell extracts from *E. coli rnh* ^+^ or Δ*rnhB* strains using mouse monoclonal antibodies raised against the α-subunit. The band intensities shown at the bottom of the gel were calculated as the percent of the band intensity observed in the *rnh* ^+^ *dinB* ^+^ strain (RI− relative intensity).
(PDF)
