# Supplementary figures and images for: Mechanisms Employed by Escherichia coli to Prevent Ribonucleotide Incorporation into Genomic DNA by Pol V
Source: PLoS Genet. 2012 Nov 8;8(11):e1003030. doi: 10.1371/journal.pgen.1003030 (PMC3493448; doi:10.1371/journal.pgen.1003030)

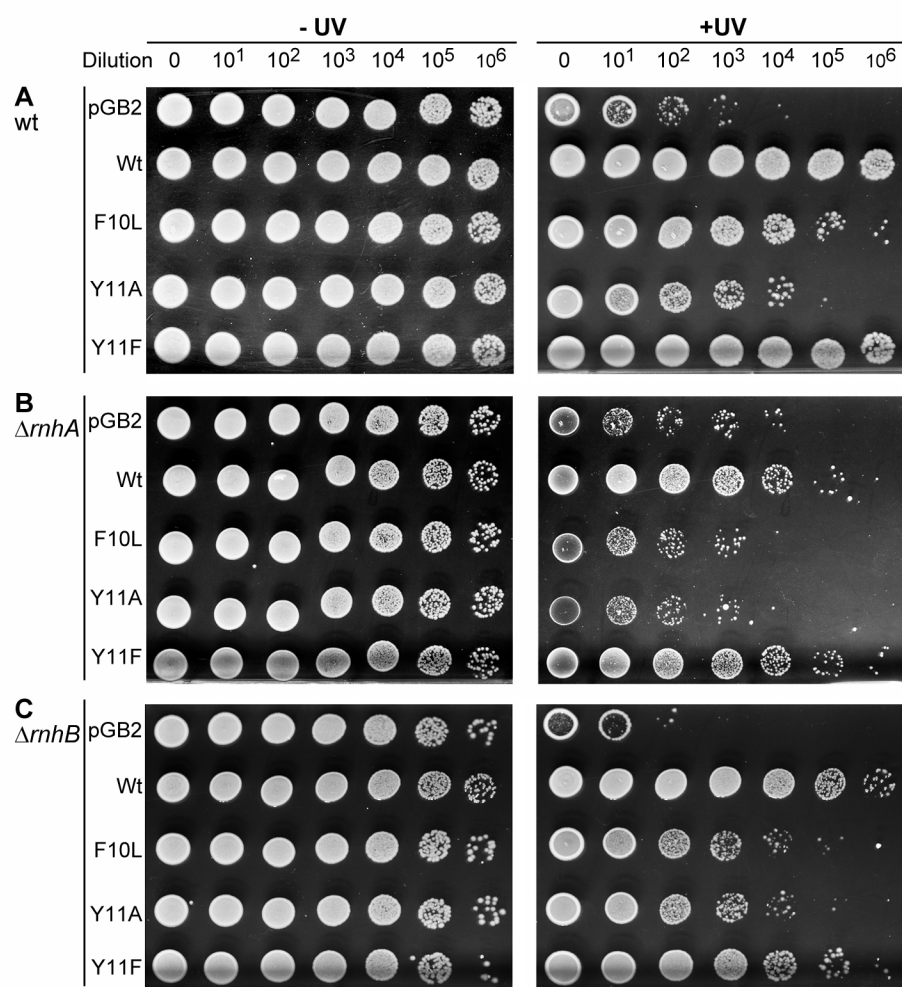

Figure S1

Supplement: Figure S1 — UV-survival of recA730 lexA(Def) ΔumuDC strains expressing pol V variants. Ten microliters of 10-fold serial dilutions of overnight cultures were spotted onto the surface of rectangular LB agar plates and exposed to 40 J/m2 (panels A and C) and 10 J/m2 (panel B) 254 nM UV-light. Both unirradiated (−) and UV-irradiated (+) plates were incubated overnight at 37°C. In each panel, UV survival is shown for the recA730 lexA(Def) ΔumuDC strains either harboring pGB2 vector, or expressing pol V variants. (PDF) [file pgen.1003030.s001.pdf]

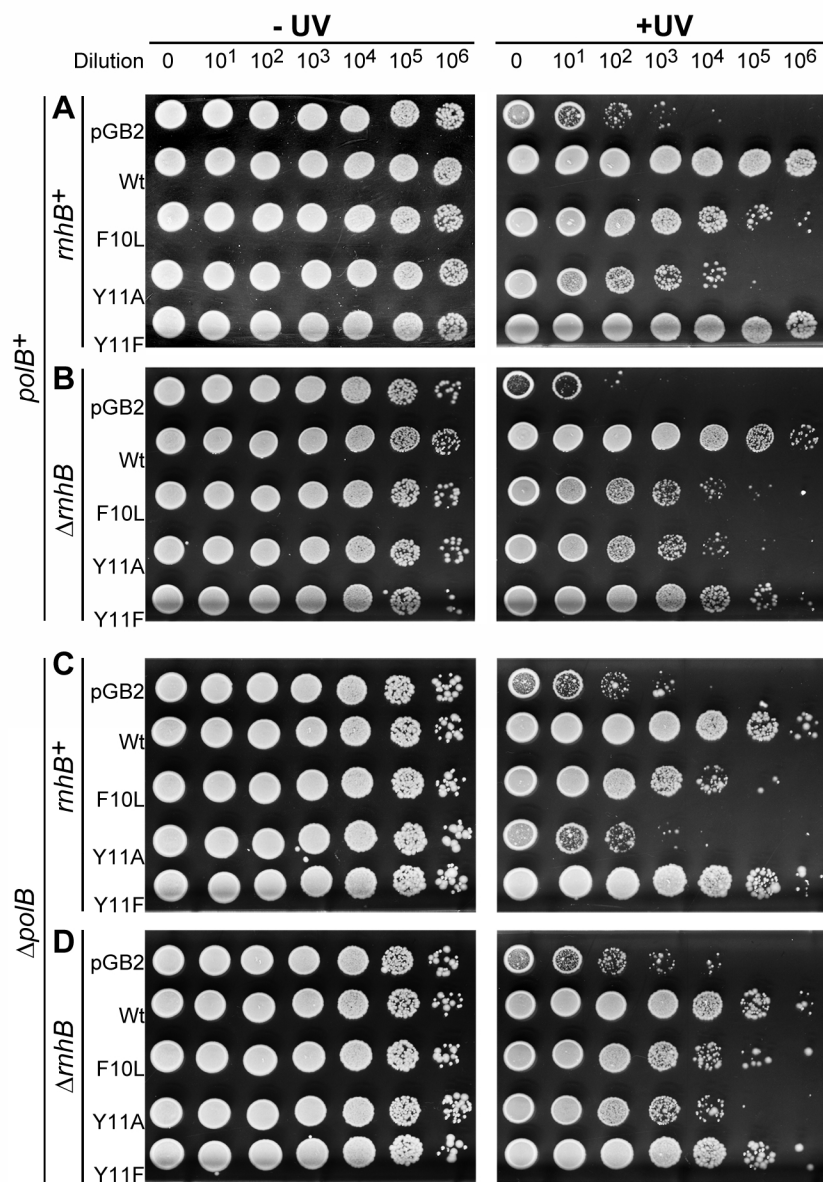

Figure S2

Supplement: Figure S2 — UV-survival of recA730 lexA(Def) ΔumuDC polB +/ΔpolB strains expressing pol V variants. Panel A and C, rnhB +; Panel B and D, ΔrnhB. Ten microliters of 10-fold serial dilutions of overnight cultures were spotted onto the surface of rectangular LB agar plates and exposed to 40 J/m2 254 nM UV-light. Both unirradiated (−) and UV-irradiated (+) plates were incubated overnight at 37°C. In each panel, UV survival is shown for the recA730 lexA(Def) ΔumuDC strains either harboring pGB2 vector, or expressing pol V variants. (PDF) [file pgen.1003030.s002.pdf]

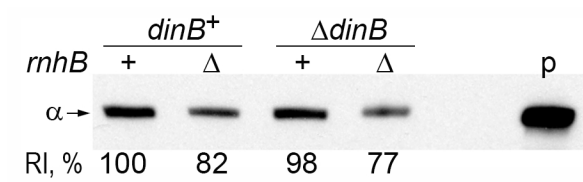

Figure S3

Supplement: Figure S3 — Western blot of the α-subunit of pol III holoenzyme in rnh + and ΔrnhB strains. The α-subunit of pol III holoenzyme was detected in whole cell extracts from E. coli rnh + or ΔrnhB strains using mouse monoclonal antibodies raised against the α-subunit. The band intensities shown at the bottom of the gel were calculated as the percent of the band intensity observed in the rnh + dinB + strain (RI− relative intensity). (PDF) [file pgen.1003030.s003.pdf]
